# Complete genomic sequence of *Vibrio fluvialis* strain IDH5335 isolated from a patient with diarrhea in Kolkata, India

**DOI:** 10.1128/MRA.00707-23

**Published:** 2023-11-09

**Authors:** Goutam Chowdhury, Kei Kitahara, Makoto Taniguchi, Kazuma Uesaka, Basilua Andre Muzembo, Debmalya Mitra, Ayumu Ohno, Thandavarayan Ramamurthy, Shanta Dutta, Shin-ichi Miyoshi, Asish Kumar Mukhopadhyay

**Affiliations:** 1 ICMR-National Institute of Cholera and Enteric Diseases, Kolkata, West Bengal, India; 2 Collaborative Research Center of Okayama University for Infectious Diseases in India at ICMR-NICED, Kolkata, West Bengal, India; 3 Graduate School of Medicine, Dentistry and Pharmaceutical Sciences, Okayama University, Okayama, Japan; 4 Oral Microbiome Center, Taniguchi Dental Clinic, Takamatsu, Kagawa, Japan; 5 Graduate School of Bioagricultural Sciences, Nagoya University, Nagoya, Aichi, Japan; Department of Biology, Queens College, Queens, New York, USA

**Keywords:** *Vibrio fluvialis*, diarrhea, bacteria, genome

## Abstract

We isolated a *Vibrio fluvialis* strain (IDH5335) from a stool sample collected from a patient with diarrhea. In this announcement, we report the complete genomic sequence of this organism, which was obtained by combining Illumina and Oxford Nanopore sequencing data.

## ANNOUNCEMENT

*Vibrio fluvialis* is a human diarrheagenic bacterium that is often isolated in India (1). Although 21 complete genomes of *V. fluvialis* have been registered to NCBI (https://www.ncbi.nlm.nih.gov/genome/browse/#!/prokaryotes/2299/, visited on 25 June 2023), most isolates were obtained outside India, prompting this report. *V. fluvialis* strain IDH5335 (2) was isolated from an acute diarrheal patient stool specimen using a thiosulfate-citrate-bile salts-sucrose (TCBS) agar plate (Eiken Chemical, Tokyo, Japan). In this process, the specimen was directly streaked onto the TCBS plate, and incubation was performed overnight at 37°C in the presence of atmospheric oxygen. Strain IDH5335, thus isolated from the plate as a yellow colony, showed positive results for both an oxidase test and string test using a 0.5% sodium deoxycholate solution. A biochemical test using the VITEK 2 GN card (bioMérieux, Marcy-l'Étoile, France) and a species-specific *V. fluvialis toxR* PCR suggested that IDH5335 belongs to *V. fluvialis* (3, 4).

To extract genomic DNA, strain IDH5335, was inoculated in Luria broth [1% (w/v) tryptone, 0.5% (w/v) yeast extract, and 1% (w/v) NaCl] and cultured at 37°C overnight. Genomic DNA was prepared from a harvested bacterial pellet using a QIAamp DNA Mini Kit (QIAGEN, Hilden, Germany) per the manufacturer’s instructions. Subsequently, the obtained genomic DNA was subjected to Illumina and Nanopore sequencing at Genome-Lead (Takamatsu, Japan) (5, 6). Default parameters were used for all software, unless otherwise specified.

For Illumina sequencing, a paired-end (2 × 150 bp) DNA library [prepared using the NEBNext Ultra II FS DNA Library Prep Kit for Illumina (NEB)] was sequenced using the NovaSeq6000 instrument. Raw sequencing data were processed using fastp v.0.19.5 (7) to trim adapters and low-quality data, yielding 3,118,979 short reads with an average length of 144.4 bp.

Long-read sequencing was performed using the GridION X5 system [Oxford Nanopore Technologies (ONT), Oxford, UK]. Unfragmented genomic DNA (1.0 µg) was used as the starting material, and small DNA molecules shorter than 10 kb were eliminated using Short Read Eliminator XS (Circulomics). The resulting DNA (600 ng) was used for library construction using a Q20 Ligation Sequencing Kit (SQK-Q20EA, ONT). The prepared library was applied to a FLO-MIN106 R10.4 flow cell (ONT). The long-read sequences were base-called using the high-accuracy base-calling mode of Guppy v.5.1.13 (ONT), generating 233,229 reads (2.2 Gb in total) with an average length of 9437.6 bp (numbers are those for reads after quality filtering, with an average Phred quality value exceeding 10.0, using NanoFilt v.2.7.1) (8).

For complete *de novo* genome assembly, both Illumina and Nanopore data were processed using Unicycler v.0.4.8 (9), followed by a final polishing step using Pilon v.1.24 (10) and manual inspection, generating two circular contigs for chromosomes 1 and 2 of 3,364,572 (49.7% GC) and 1,465,818 bp (50.2% GC), respectively, and other circular but smaller contigs for plasmids with lengths of 370,014 (44.7% GC), 103,088 (45.0% GC), and 83,499 bp (48.4% GC, Table 1). We confirmed that no circular contigs featured structural mis-assembly using SV-Quest (K. Uesaka, unpublished data, https://github.com/kazumaxneo/SV-Quest). This process was repeated with different +1 positions. Automatic annotation was performed using the annotation pipeline DFAST v.1.2.0 (11) provided by the DNA Data Bank of Japan (DDBJ), which predicted 5,017 coding sequences, 31 rRNA genes, and 107 tRNA genes. A digital DNA-DNA hybridization (dDDH) analysis using the Type Strain Genome Server (12, 13) showed that strain IDH5335 and *V. fluvialis* NBRC 103150 (the type strain of *V. fluvialis*) had a dDDH (d_4_) value of 87.6%, suggesting that strain IDH5335 certainly belongs to *V. fluvialis* members at species level [note that the threshold value of dDDH (d_4_) for species-level identification is 70%] (12–14). Although researchers in India tend to focus more, for example, on the cholera-causing pathogen *V. cholerae* O1/O139 (15) and shigellosis-causing pathogen *Shigella* spp. (16) , *V. fluvialis*, which is paid lesser attention, has also been recognized as a significant emerging pathogen in India (1, 4). It is anticipated that more and more genomic sequences of *V. fluvialis* strains circulating in India will be determined and genomic and epidemiological features of *V. fluvialis* will be finely clarified.

**TABLE 1:** Statistics of the assembled genome of *Vibrio fluvialis* strain IDH5335

Name	Length (bp)	Circular/linear	GC (%)	Average read depth
Chromosome 1	3,364,569	Circular	49.7	148.6
Chromosome 2	1,465,818	Circular	50.2	144.2
Plasmid pIDH5335-1	370,014	Circular	44.7	281.8
Plasmid pIDH5335-2	103,088	Circular	45.0	625.2
Plasmid pIDH5335-3	83,499	Circular	48.4	269.9

## Data Availability

The complete chromosomal and plasmid sequences were deposited at DDBJ/EMBL/GenBank under the accession numbers AP028128 (chromosome 1), AP028129 (chromosome 2),AP028130 (plasmid 1), AP028131 (plasmid 2), andAP028132 (plasmid 3). Raw sequencing data were deposited in the DDBJ SRA database under the accession numbers DRR464391 (Illumina) and DRR464393 (Nanopore).
